# Navigating specific targets of psychoneurological symptom cluster in breast cancer: a computer-simulated network analysis

**DOI:** 10.3389/fonc.2026.1869133

**Published:** 2026-06-26

**Authors:** Jiyu Cai, Zhao Liu, Xianliang Liu, Chunzi Wan, Xia Duan

**Affiliations:** 1 Nursing Department, Shanghai Key Laboratory of Maternal Fetal Medicine, Shanghai Institute of Maternal-Fetal Medicine and Gynecologic Oncology, Shanghai First Maternity and Infant Hospital, School of Medicine, Tongji University, Shanghai, China; 2 Department of Thyroid and Breast Surgery, The Affiliated Hospital of Xuzhou Medical University, Xuzhou, China; 3 School of Nursing and Health Studies, Hong Kong Metropolitan University, Hong Kong, Hong Kong SAR, China

**Keywords:** breast cancer, computer-simulated interventions, dynamic symptom interactions, network analysis, psychoneurological symptom cluster

## Abstract

**Objectives:**

Sleep disturbance, cancer-related fatigue, emotional distress (anxiety and depression), and pain, collectively known as psychoneurological symptom cluster (PNSC), were common during and after anti-cancer treatment in breast cancer patients. This study aimed to explore the relationship among PNSC to identify potential intervention targets.

**Methods:**

In this cross-sectional study, 304 breast cancer patients who received treatment in the Breast Surgery Department from June 2025 to January 2026 were recruited. Self-report data were collected using the Cancer Fatigue Scale, the Pittsburgh Sleep Quality Index, Hospital Anxiety and Depression Scale, and Visual Analog Scale for Pain. Static symptom interrelationships were examined using Gaussian network model, and putative directional associations were explored using Bayesian network analysis. The dynamic correspondence between symptoms was explored through computer-simulated interventions, and simulation-derived candidate symptom targets were identified.

**Results:**

The network analysis identified physical fatigue, sleep latency, and depression as core symptoms, with expected influence (EI) values of 1.883, 0.940, and 0.794, respectively; furthermore, the study identified physical fatigue, pain, and daytime dysfunction as bridging symptoms, with bridge expected influence (bEI) values of 1.824, 1.558, and 1.331, respectively. The estimated Bayesian network also suggested that physical fatigue occupied as a key node within the network. The results of computer-simulated interventions showed that depression produced the largest reduction, with sum scores declining from 5.98 to 4.67; this was followed by the affective fatigue and physical fatigue. Meanwhile, poor sleep efficiency was associated with an increase in the total sum score, from 5.99 to 6.04.

**Conclusions:**

Our findings suggest that physical fatigue and depression may be prioritized as intervention targets to break the vicious cycle of symptom interactions within the PNSC. The integration of computer-simulated interventions and traditional network analysis allowed for prioritizing the effects of individual symptoms at both static and dynamic levels, thereby improving the accuracy and effectiveness of targeted interventions. Further research is needed to verify whether targeting these symptoms can improve clinical outcomes.

## 1 Introduction

Breast cancer represents a significant and growing global health challenge, with projections estimating 3.2 million new cases and 1.1 million deaths worldwide by 2050 (1). Patients with breast cancer may experience severe disease-and treatment-related symptoms; notably, evidence indicates the existence of one PNSC composed of cancer-related fatigue, emotional distress (anxiety and depression), sleep disturbance, and pain (2). Affecting more than 50% of breast cancer patients (3), this cluster has a profound and lasting impact on quality of life, with symptoms persisting for years after treatment completion. Despite their severe impact and persistence up to years after the treatment completion, these symptoms often remain inadequately diagnosed or managed in clinical practice (4, 5).

Compared to a single symptom, PNSC not only have negative effects on themselves, but also develop synergistically and reinforce each other, thereby exacerbating functional impairments and reducing treatment adherence in breast cancer patients (6). The co-occurrence presentation of PNSC symptoms suggested that they may share a common underlying mechanism, including inflammatory responses, proinflammatory cytokines, hypothalamic-pituitary-adrenal axis (7, 8); however, the exact mechanism of their occurrence and development remains unclear. Existing evidence suggests that the strength of the relationships between different symptoms within the PNSC, as well as the core and bridging symptoms, may vary depending on the cancer type and evolves dynamically with disease progression (6, 9). The formation and interrelationship mechanisms of PNSCs in breast cancer patients remain unclear. Current studies mainly analyze the relationship between symptoms from a holistic perspective, rather than analyzing the relationships between them from the perspective of internal factors of symptoms (10). Therefore, more rigorous research is needed to assess the presence and internal composition of PNSC in breast cancer patients (8).

In 2017, the previous study (11) proposed the network theory of mental disorders, which posits that the development and maintenance of mental illness are driven by potential interactions among various symptoms. When these potential relationships are sufficiently strong, they form a feedback loop that allows symptoms to sustain and exacerbate one another. Alterations within this network can thereby give rise to a persistent pathological state. Network analysis is an innovative approach that can quantify the complex association between PNSC symptoms and identify core symptoms. More cost-effective interventions can then be designed to target these core symptoms, thereby impacting the entire symptom cluster (12). The previous study (13) conducted a network analysis of cancer-related fatigue in breast cancer patients, revealed that depression was closely related to all dimensions except mental fatigue, and was the core symptom in the network. Li et al. (14) investigated the interaction between cancer related fatigue and sleep in breast cancer patients. The results showed that sleep quality was the strongest predictor of cancer-related fatigue, and daytime dysfunction in sleep quality had the strongest bridging strength. The network structure of PNSC varies depending on the study population, cancer progression stage, and research methodology employed. Consequently, no consensus has yet been reached regarding the core symptoms of PNSC among patients with breast cancer.

Furthermore, we need to identify intervention targets within the symptoms of PNSC. Computer-simulated intervention aims to find the most effective symptom intervention targets by altering the activation probability of target symptoms. Unlike traditional clinical interventions, computer-simulated intervention can simulate various real-world scenarios and generate dynamic network systems, thus overcoming the limitations of traditional network models (15). In preliminary trials using computer simulations, target symptom was inactivated by setting the activation probability to zero, thereby assessing the impact of these symptoms on overall network activation and precisely identifying which symptoms have the greatest impact on the overall activation level (16). Therefore, computer-simulated intervention provides crucial information for precise guidance of symptom management.

The aim of study was to integrate traditional network models (Gaussian network analysis and Bayesian network) with computer-simulated interventions for a deep understanding of the internal network structure and dynamic interactions of PNSC, ultimately revealing the potential intervention target.

## 2 Methods

### 2.1 Participants

This study used convenience sampling (17) to recruit 304 breast cancer patients from the breast surgery departments of a tertiary hospital in Xuzhou, Jiangsu Province, between August 2025 and January 2026. The inclusion criteria were as follows: (1) a confirmed pathological diagnosis of primary breast cancer, (2) aged over 18 years, (3) participants voluntarily agreed to participate in the study after being invited. The exclusion criteria included an inability to complete self-report assessments. The research protocol was approved by the Ethics Committee of Affiliated Hospital of Xuzhou Medical University (XYFY2025-KL420-01).

All researchers received standardized training and systematically screened potential participants via the hospital’s medical workstation system. Eligible patients who provided informed consent to participate were invited to a quiet room, where a field survey was administered using a combination of semi-structured interviews and self-completed questionnaires. Participants were also informed that they had the right to refuse to answer any questions or withdraw from the study at any time, and that such actions would not affect their subsequent treatment or care in any way. To protect the rights and interests of patients, all personal information collected in this survey will be kept strictly confidential.

### 2.2 Measures

Based on previous study (18), demographic data were collected including age, sex, education, employment, residence, marital status, menstrual status and the stage of the breast cancer.

Cancer-related fatigue was measured using the Cancer Fatigue Scale (CFS) (19), a 15-item self-report tool designed to evaluate three key dimensions of fatigue: physical fatigue, affective fatigue, and cognitive fatigue. CFS used on a scale of 0 (not at all) to 4 (very much), with a total score ranging from 0 to 60 points. Higher scores indicated more sever fatigue. Previous studies had confirmed the CFS has good psychometric properties1, and the Chinese version of CFS had also been proven to have satisfactory reliability and validity (20, 21).

The Pittsburgh Sleep Quality Index (PSQI) was developed by Buysse et al. (22) to evaluate subjective sleep disturbance over the past month. This index consisted of 19 self-rating items that can be categorized into seven domains: sleep quality, sleep latency, sleep duration, sleep efficiency, sleep disturbance, medication use, and daytime dysfunction. Each item was scored on a 4-point Likert scale (0-3). The total core ranged from 0 to 21, and higher scores on this scale indicated poorer subjective sleep quality. The Chinese version had been validated and well adapted (23).

Anxiety and depression were measured using the Hospital Anxiety and Depression Scale (HADS) (24). This index included 14-item self-report questionnaire, which seven items measured symptoms of anxiety and seven items measure symptoms of depression. Each item was scored on a 4-point Likert scale (0-3). The total core of each scaled ranged from 0 to 21, and higher scores on this scale indicated poorer psychological status.

Pain intensity was assessed using the Visual Analogue Scale (VAS). VAS consists of a 10-cm horizontal scale, with 0 on the left end indicating “no pain”, and 10 on the right end indicating the “worst pain”. correspond to greater pain intensity compared with baseline (25). The VAS has been validated as a valid, reliable, concise and easily interpretable outcome measure.

### 2.3 Statistical analysis

All data analysis were performed in R 4.5.2.

Descriptive statistics were calculated, including frequencies, percentages, means and standard deviations.

First, a Gaussian network analysis aimed at exploring the correlations between PNSC symptoms and identifying the most central and bridge symptoms. The extended Bayesian information criterion (EBIC) model and the graphical Gaussian model (GGM) with the visual least absolute shrinkage and selection operator (LASSO) were used to determine the correlations among PNSC symptoms. The line connecting two nodes was an edge, and each node in the network model represents a particular symptom. EI was used to quantify central symptoms, and referred to the highly influential symptoms in the network (26). Meanwhile, bEI was used to quantify the bridge symptoms, and referred to symptoms that act as connecting points between different symptom clusters (27). The non-parametric bootstrap was used to assess the accuracy of edges. The narrower constructed confidence interval (CI) indicated a trustworthy network. Correlation stability (CS) coefficient was used to assess the stability of the network. Generally, the value of CS coefficient should exceed 0.5 but be no less than 0.25 (28).

Second, a Bayesian network analysis was utilized to identify directional associations among PNSC. Directed edges represent a direct or indirect link. If symptom X consistently precedes symptom Y, X may be the cause of Y. The hill-climbing algorithm was employed to infer the network structure, and connections that consistently exceed the preset threshold for both edges and edge direction in multiple iterations are retained (29, 30).

Third, computer-simulated interventions were conducted to identify the most efficient, the symptom-specific targets. Utilizing the NodeIdentifyR algorithm (NIRA) based on the Gaussian Model, two intervention types were simulated: alleviating intervention and aggravating intervention (31, 32). Specifically, NIRA intervened by systematically adjusting the threshold parameters of various symptoms and generated 15,000 simulated “participants” to assess the effectiveness of the intervention. The intervention intensity was adjusted by modifying the standard deviation of the estimated threshold. In this study, the threshold was adjusted by plus and minus two standard deviations (33).

## 3 Results

Among the 304 participants, one was a male and the remaining 303 were females, with a mean age of 49.39 years (SD = 10.03). The majority of patients, 284 (93.4%) were married, and 125 (41.1%) resided in rural areas. The average score for cancer-related fatigue was 19.7 (SD = 9.91); the average score for anxiety was 5.20 (SD = 3.77); the average score for depression was 4.04 (SD = 3.63); the average score for sleep was 5.50 (SD = 3.38); and the average score for pain was 2.09 (SD = 2.14). Additional details were showed in **Table 1**.

**Table 1:** Demographic analysis among patients diagnosed with breast cancer.

Variables	n(%)
Age, (mean ± SD)	49.39 ± 10.03
Sex	
Female	303 (99.7)
Male	1 (0.3)
Educational attainment	
Secondary school or below	207 (68.1)
High school	43 (14.1)
University or above	54 (17.8)
Employment status	
Employed	100 (32.9)
Unemployed	164 (53.9)
Retired	40 (13.2)
Residence	
City	114 (37.5)
Town	65 (21.4)
Countryside	125 (41.1)
Marital status	
Single	5 (1.6)
Married	284 (93.4)
Divorced	7 (2.3)
Widowed	8 (2.6)
Menstrual status	
Regular	115 (37.8)
Irregular	7 (2.3)
Postmenopausal	171 (56.3)
NA	1 (0.3)
Stage of disease	
I	69 (22.7)
II	180 (59.2)
III	55 (18.1)
Cancer-related fatigue(mean ± SD)	
Physical fatigue	8.94 ± 5.39
Affective fatigue	5.98 ± 3.40
Cognitive fatigue	4.77 ± 3.40
Emotional distress (mean ± SD)	
Anxiety	5.20 ± 3.77
Depression	4.04 ± 3.63
Sleep (mean ± SD)	
Sleep quality	0.89 ± 0.83
Sleep latency	1.42 ± 0.72
Sleep duration	0.83 ± 0.93
Sleep efficiency	0.81 ± 1.06
Sleep disturbance	0.95 ± 0.43
Medication use	0.05 ± 0.35
Daytime dysfunction	0.55 ± 0.71
**Pain (mean ±SD)**	2.09 ± 2.14

### 3.1 Gaussian network analysis

**Figure 1:**
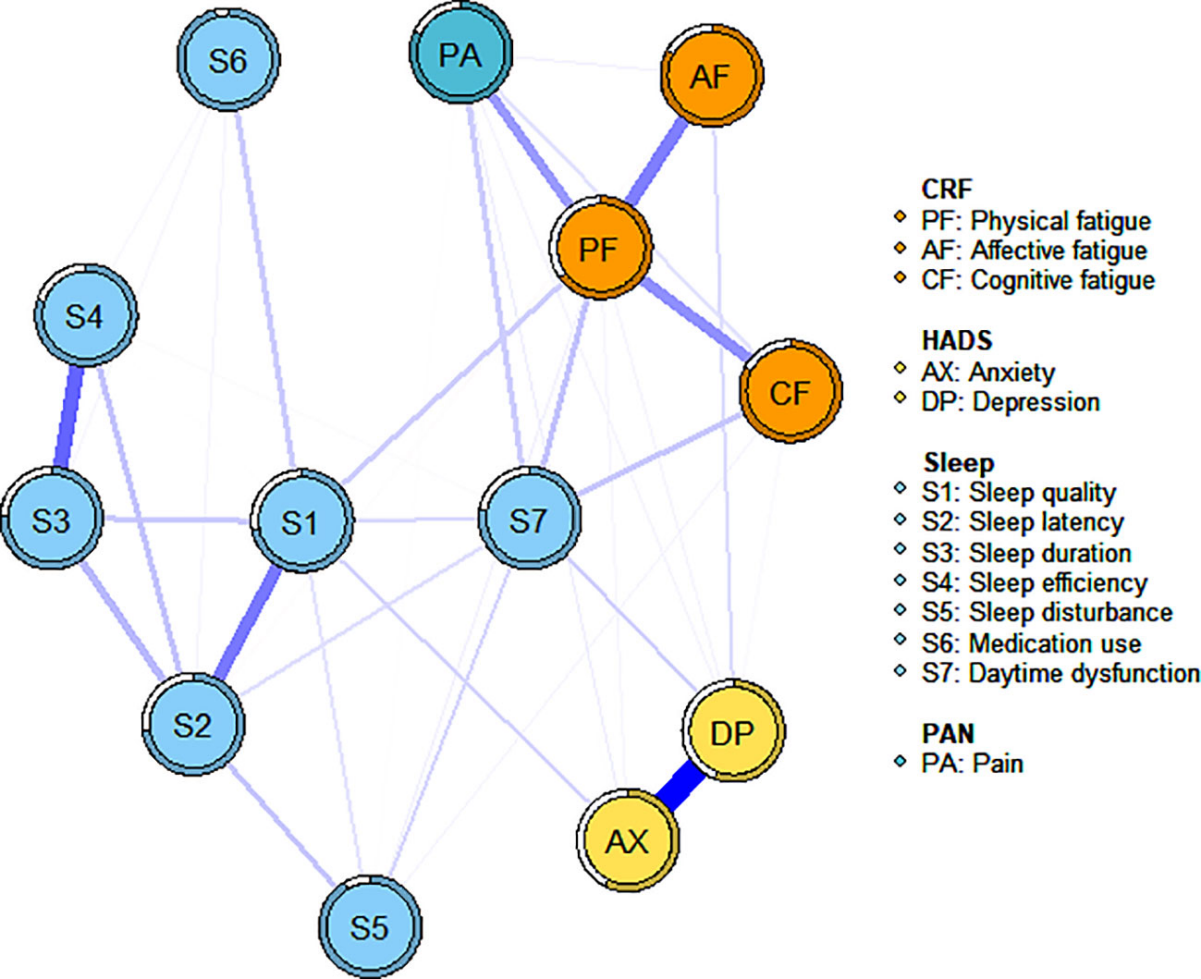
Gaussian network analysis visualization and centrality metrics for psychoneurological symptom cluster.

Associations between PNSC were visualized using a Gaussian network model. After removing edges with too little correlation through the penalization factor, the network showed 39 (50.0%) non-zero edges out of a possible 78 potential edges. Except for the negative correlation between affective fatigue and sleep latency, all others represented positive associations. The network structure of PNSC among breast cancer patients is illustrated in **Figure 1**. A circular pie chart around each node was used to represent node predictability. The average predictability was 0.761 (ranging from 0.559 to 0.960), meaning that the surrounding nodes could typically explain 76.1% of each node’s variance.

**Figure 2:**
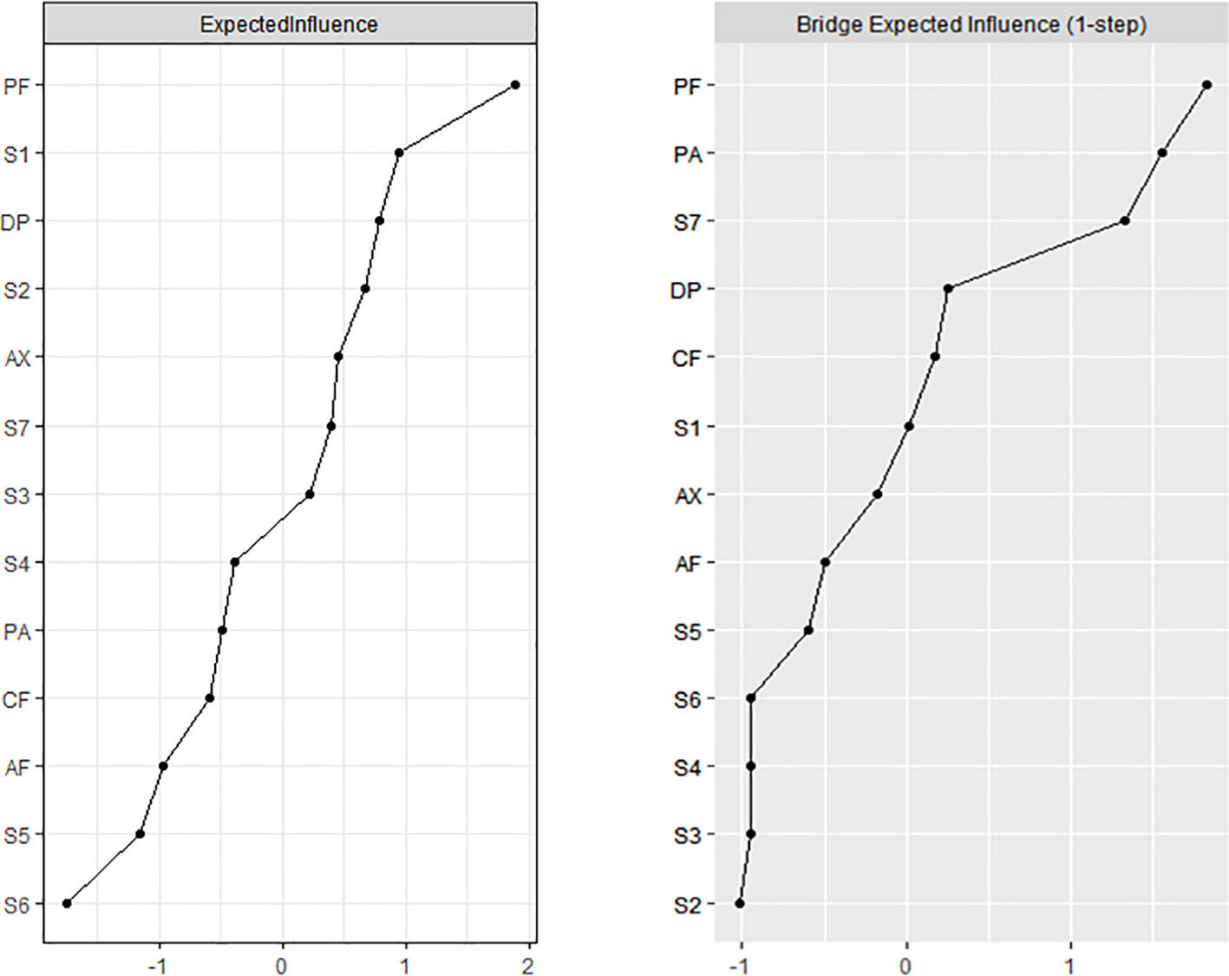
Centrality indices: expected influence and bridge expected influence values.

The EI and bEI values of symptoms in the PNSC network were shown in **Figure 2**. The most central symptom was physical fatigue, followed by sleep latency and depression, with EI values of 1.883, 0.940, and 0.794, respectively (**Figure 2**, left). To understand the network of PNSC symptoms in breast cancer patients, it is crucial to understand these three symptoms. The main symptoms bridging the PNSC communities were physical fatigue, pain, and daytime dysfunction, with bEI values of 1.824, 1.558, and 1.331, respectively (**Figure 2**, right part). These symptoms play a crucial role in linking PNSC within the network.

**Figure 3:**
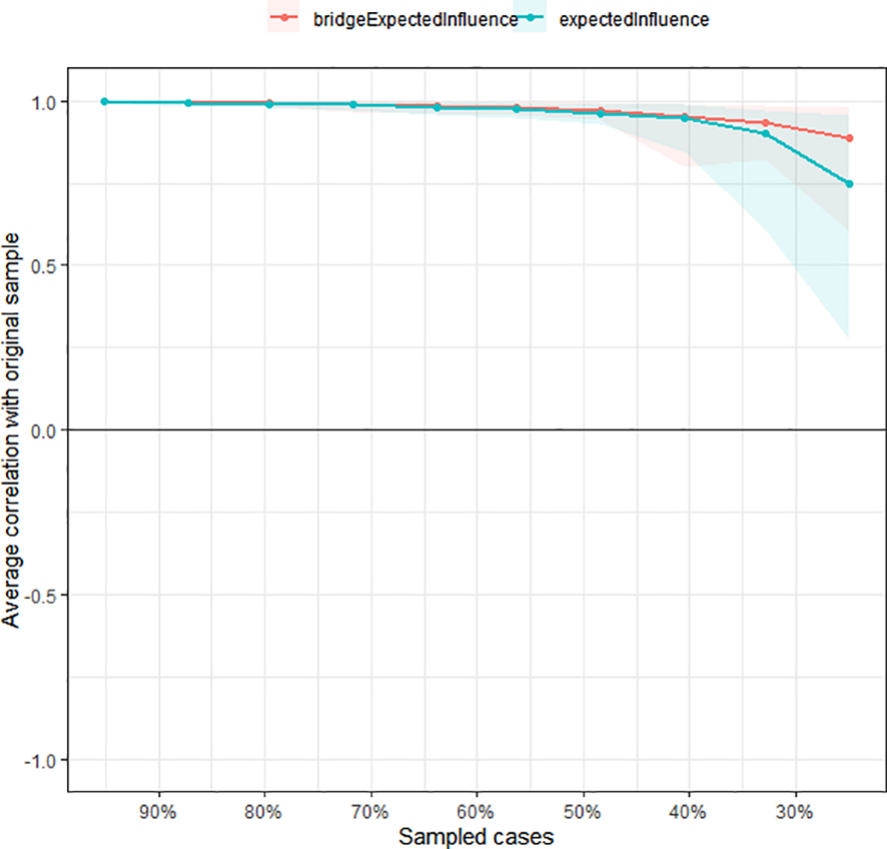
The stability of centrality and bridge centrality indices according to a case-dropping bootstrap method.

The symptoms in the PNSC network had good stability. **Figure 3** showed that the stability of the EI CS-coefficient was 0.671, indicating that the network did not significantly change when 67.1% of the sample was dropped. The bEI CS-coefficient was 0.671. The central symptoms were significantly different from the other nodes, according to the bootstrapped stability test for EI (**Supplementary Figure 1**). **Supplementary Table 1** and **Supplementary Figure 2** showed the estimated CIs of edge weights by bootstrapping. The relatively narrow CIs indicate acceptable accuracy.

### 3.2 Bayesian network model

**Figure 4:**
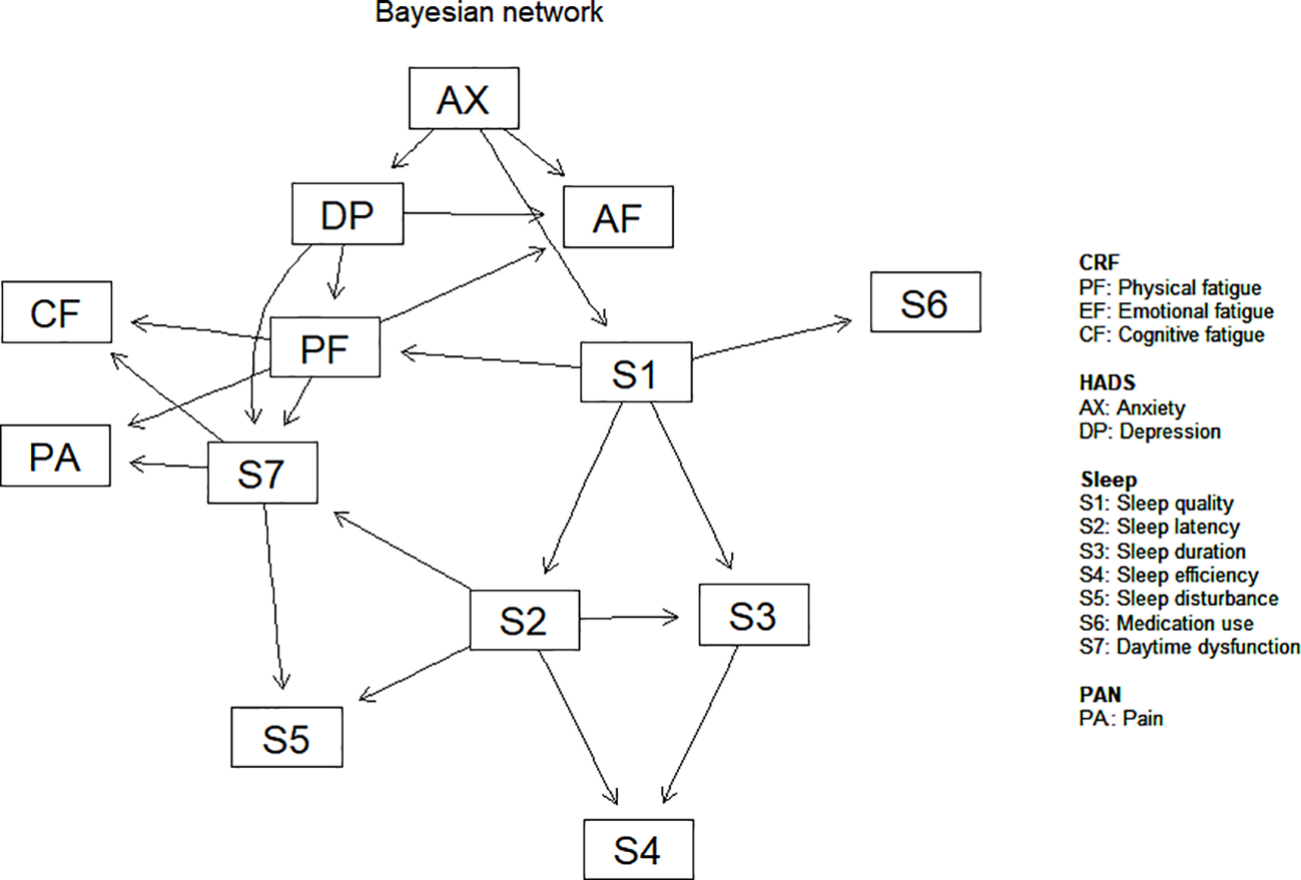
Depiction of the Bayesian network model of symptoms.

Directed edges represent potential causal relationships, 22 directed edges were preserved. Due to its position at the top of the figure, anxiety was identified as the parent node, highlighting its central role in symptom interactions. Anxiety showed directed connections with depression, sleep quality, and affective fatigue, and was also indirectly connected with other nodes. Physical fatigue showed a strong directed positive association with sleep quality(β=1.959) and also positively associated with depression (β=0.454); this pattern suggested a directed pathway in which anxiety may be linked to physical fatigue through depression and/or sleep quality. Physical fatigue also served as an important intermediate connecting multiple functional modules. Physical fatigue showed directed positive association with affective fatigue (β=0.314) and cognitive fatigue (β=0.281), a weak directed association with daytime dysfunction (β=0.048), and a positive association with pain (β=0.175). In summary, physical fatigue exhibited the most interrelationships within the network and was the bridge symptom. Due to their position in the key directed pathways and its central role across symptom domains, physical fatigue was identified as a key node in the Bayesian network. The Bayesian network model was shown in **Figure 4**.

### 3.3 Effects of computer-simulated interventions

**Figure 5:**
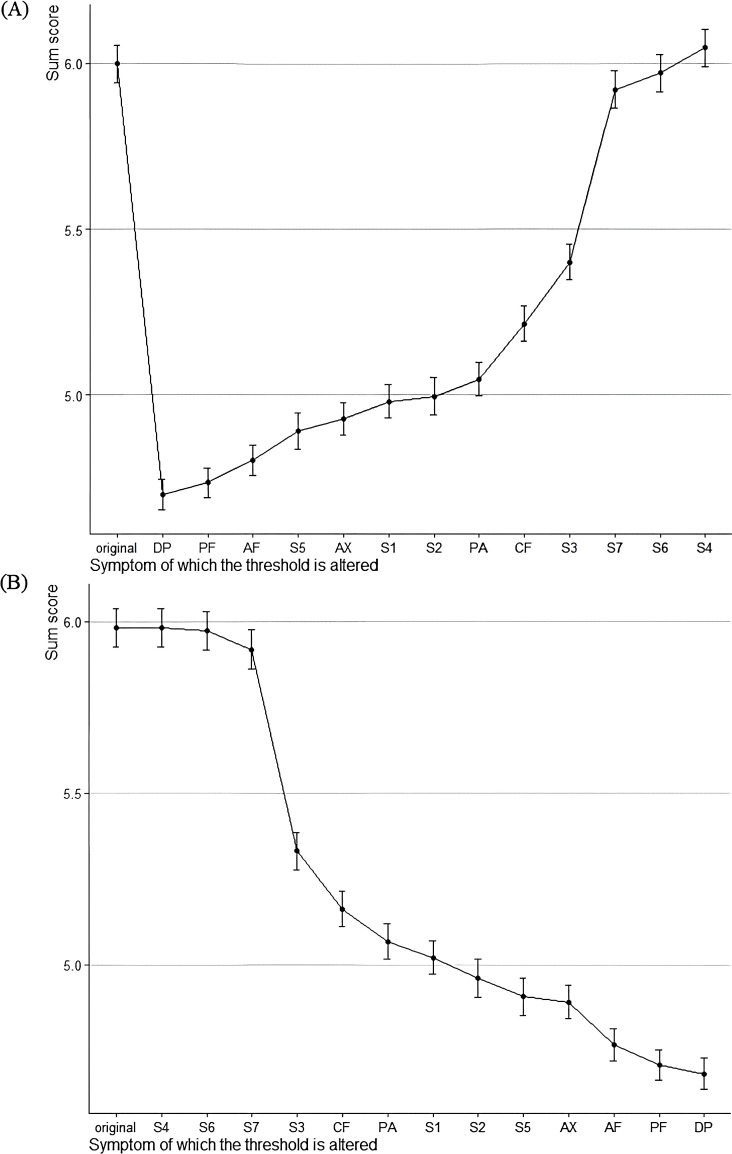
The effect of computer-simulated interventions on the symptoms. **(A)** Projected Effects of alleviating intervention depicted by sum score of target symptoms. **(B)** Projected Effects of aggravating intervention depicted by sum score of target symptoms.

The results of the computer-simulated intervention were shown in **Figure 5**. The results showed that depression had the lowest sum score during the computerized alleviated intervention, suggesting that depression was the best-alleviated target for this study with sum scores declining from 5.98 to 4.67. This 1.31-point reduction suggested a significant decrease in overall symptom levels within the network. Moreover, affective fatigue can reduce the overall network score from 5.98 to 4.75; Physical fatigue, as the central symptoms, can reduce the overall score from 5.98 to 4.79. These two nodes may be important candidate targets for reducing overall symptom burden in computer-simulated intervention. In addition, simulated aggravation interventions were conducted. Sleep efficiency significantly increased the overall symptom levels within the network with sum scores rising from 5.99 to 6.04. This increase of 0.05 suggested that sleep efficiency may warrant further investigation as a potential target for preventing symptom aggravation. Overall, aggravating interventions yielded more moderate overall changes.

## 4 Discussion

Taking the intrinsic constitutive characteristics of symptoms as an entry point, this study integrated network analysis with computer-simulated interventions to explore potential symptom targets for PNSC in breast cancer patients. The study indicated that the core symptoms were physical fatigue, sleep latency, and depression, while the bridge symptoms were physical fatigue, pain, and daytime dysfunction. In the Bayesian network analysis, physical fatigue appeared to be a key node within the network. The results of computer-simulated interventions showed that depression produced the largest reduction in the overall network score under the simulated alleviation intervention, followed by affective fatigue and physical fatigue. Meanwhile, sleep efficiency may warrant further investigation as a potential target for preventing PNSC symptom aggravation.

The findings suggested that symptoms exerting a significant influence within traditional network models also showed greater reactivity to computer-simulated intervention. However, the quantified results of the computer-simulated interventions were not completely consistent with the central and bridge symptoms identified by traditional network analysis models. Previous study (33) reported that the most effective targets identified by computer-simulated intervention were related to the most central nodes in static network structures but were not always exactly the same, which was consistent with our findings. The structure of traditional network models may fail to capture the complexity of the network, whereas the dynamic symptom relationships captured by computer simulations may provide a more nuanced perspective. Therefore, relying solely on network structural indicators (EI and bEI) cannot directly determine the effective intervention target symptoms of PNSC in breast cancer patients; instead, a comprehensive assessment incorporating dynamic computer-simulated intervention analyses was required to facilitate targeted symptom management.

In the Gaussian network structure analysis, physical fatigue and depression both exhibited high EI values, suggesting that they occupied relatively central positions in the estimated symptom network. The computer-simulated interventions analyses indicated that depression and physical fatigue were associated with relatively large reductions in the overall network activation level during simulated alleviation interventions. Consequently, physical fatigue and depression were attributes that served as both central symptoms in the network structure and key intervention targets in the dynamic system, playing a crucial role in the dynamic changes of PNSC. Numerous previous studies have indicated that fatigue exhibits the strongest association with the severity of a wide range of physiological and psychological symptoms (34, 35). The underlying pathway may involve systemic inflammatory responses mediated by pro-inflammatory cytokines, which subsequently lead to the exacerbation of other symptoms (36). Fatigue manifests across different dimensions; the patterns of physical fatigue differ from those of affective fatigue. Yet, currently, there is a scarcity of research specifically focused on physical fatigue. The physical fatigue in breast cancer patients may be related to disruptions in the circadian cortisol rhythm, and the decline in cardiopulmonary function after treatment may also contribute to physical fatigue (37, 38). Physical fatigue may represent a clinically relevant symptom domain connecting physiological and psychological adaptation. Therefore, focusing on the precise assessment and targeted intervention of physical fatigue may be more cost-effective than the broader management of cancer-related fatigue.

A meta-analysis review (39) specifically regarding multidimensional cancer-related fatigue in breast cancer patients indicated that exercise interventions produced moderate improvements in physical fatigue. In contrast, psychological therapies and alternative therapies (such as acupressure, bright light and multivitamin) showed non-significant on physical fatigue. The beneficial effects of exercise on physical fatigue were observed not only in patients undergoing active treatments but also in breast cancer survivors. Therefore, individualized exercise regimens targeting physical fatigue may serve as a crucial strategy for the prevention and management of physical fatigue in breast cancer patients. The relevant studies reported that exercise interventions combining aerobic and resistance training have been suggested to be effective (40). However, as the optimal forms, duration, and intensity of exercise, as well as strategies to enhance adherence, remain unclear, highlighting the need for further research to establish precise, feasible, and clinically effective exercise protocols.

Depression in computer simulation interventions has surpassed the core symptom physical fatigue initially emphasized by Gaussian network analysis. Among all types of cancer patients, the depression prevalence in breast cancer patients is the third highest, with the incidence estimated to be between 10% and 30% (41). It has a negative impact on women’s treatment, quality of life, and self-care. During the treatment process, depressive symptoms often overlap with physical symptoms caused by the disease or treatment, so the diagnosis of depression in cancer patients is often overlooked during treatment (42). Therefore, regularly monitoring the progression of depression is crucial, as it helps in developing personalized interventions that address its multifaceted impacts.

Computer simulation interventions suggested that sleep efficiency may be the potential healthcare target for preventing PNSC symptom aggravation. In the sleep evaluation indicators of breast cancer patients, sleep efficiency is less frequently mentioned. Sleep efficiency is defined as the ratio of actual sleep time to time spent in bed and is an objective indicator of sleep quality (43). Sleep efficiency is more strongly correlated with good sleep than sleep continuity variables such as sleep onset latency, final wake time, and wake time after sleep onset (44). Hasler et al. (45) suggested that the higher the sleep efficiency, the more complete the sleep. Polysomnography is considered the gold standard for detecting sleep efficiency. With the popularization of electronic devices, devices like fitness trackers can also monitor sleep efficiency closely. Measures such as medications, acupuncture, and acupoint massage can also effectively improve sleep efficiency.

### 4.1 Limitations

This study had certain limitations. First, the recruitment of study sample used convenience sampling which limited the external validity of the findings. Future multi-center studies with larger and more diverse samples are needed to validate the stability and generalizability of the findings. Second, the design of cross-sectional cannot directly assess the temporal dynamics and causal relationships among symptoms, longitudinal research might be more appropriate for future studies (46). Third, this study provided a new perspective on intervention targets, the gap between these findings derived from computer-simulated interventions and real-world interventions remained unclear. Therefore, longitudinal studies or intervention trials are needed to compare the results of computer-simulated interventions with those of real-world interventions to further verify the feasibility of simulation-based results. Fourth, this study focused on symptom nodes, potentially overlooking the significant influence of factors. Future study should incorporate additional non-symptom nodes to enhance our comprehensive understanding of symptom networks.

## 5 Conclusions

Physical fatigue constituted the most central symptom within the entire symptom network, serving also as a bridging symptom. Depression was the potential target for intervention in terms of symptom alleviation, while simultaneously ranking as the third most central symptom. By integrating computer-simulated interventions with traditional network analysis methods, symptoms can be prioritized based on their static and dynamic influence, thereby significantly improving the accuracy and effectiveness of clinical interventions. Future studies should be conducted in in larger-scale, multi-center cohorts of breast cancer patients with diverse demographic characteristics.

## Data Availability

The data that support the findings of this study are available on request from the corresponding author. The data are not publicly available due to privacy or ethical restrictions.

## References

[1] Kim J Harper A McCormack V Sung H Houssami N Morgan E . Global patterns and trends in breast cancer incidence and mortality across 185 countries. Nat Med. (2025) 31:1154–62. doi: 10.1038/s41591-025-03502-3 39994475

[2] Grégoire C Baussard L Ernst M Diep A Faymonville ME Devos M . Evaluation of a psychoneurological symptom cluster in patients with breast or digestive cancer: a longitudinal observational study. BMC Cancer. (2024) 24:51. doi: 10.1186/s12885-023-11799-x 38195471 PMC10777491

[3] Albusoul RM Berger AM Gay CL Janson SL Lee KA . Symptom clusters change over time in women receiving adjuvant chemotherapy for breast cancer. J Pain Symptom Manage. (2017) 53:880–6. doi: 10.1016/j.jpainsymman.2016.12.332 28062343 PMC5410185

[4] Abrahams HJG Gielissen MFM Verhagen C Knoop H . The relationship of fatigue in breast cancer survivors with quality of life and factors to address in psychological interventions: A systematic review. Clin Psychol Rev. (2018) 63:1–11. doi: 10.1016/j.cpr.2018.05.004 29852324

[5] Hamer J McDonald R Zhang L Verma S Leahey A Ecclestone C . Quality of life (QOL) and symptom burden (SB) in patients with breast cancer. Support Care Cancer. (2017) 25:409–19. doi: 10.1007/s00520-016-3417-6 27696078

[6] de Rooij BH Oerlemans S van Deun K Mols F de Ligt KM Husson O . Symptom clusters in 1330 survivors of 7 cancer types from the PROFILES registry: A network analysis. Cancer. (2021) 127:4665–74. doi: 10.1002/cncr.33852 34387856 PMC9291877

[7] Doong SH Dhruva A Dunn LB West C Paul SM Cooper BA . Associations between cytokine genes and a symptom cluster of pain, fatigue, sleep disturbance, and depression in patients prior to breast cancer surgery. Biol Res Nurs. (2015) 17:237–47. doi: 10.1177/1099800414550394 25304131 PMC5486211

[8] Kim HJ Barsevick AM Fang CY Miaskowski C . Common biological pathways underlying the psychoneurological symptom cluster in cancer patients. Cancer Nurs. (2012) 35:E1–e20. doi: 10.1097/NCC.0b013e318233a811 22228391

[9] Lin Y Bruner DW Paul S Miller AH Saba NF Higgins KA . A network analysis of self-reported psychoneurological symptoms in patients with head and neck cancer undergoing intensity-modulated radiotherapy. Cancer. (2022) 128:3734–43. doi: 10.1002/cncr.34424 35969226 PMC9529994

[10] Chartogne M Rahmani A Landry S Bourgeois H Peyrot N Morel B . Neuromuscular, psychological, and sleep predictors of cancer-related fatigue in cancer patients. Clin Breast Cancer. (2021) 21:425–32. doi: 10.1016/j.clbc.2020.12.002 33422432

[11] Borsboom D . A network theory of mental disorders. World Psychiatry. (2017) 16:5–13. doi: 10.1002/wps.20375 28127906 PMC5269502

[12] Schellekens MPJ Wolvers MDJ Schroevers MJ Bootsma TI Cramer AOJ van der Lee ML . Exploring the interconnectedness of fatigue, depression, anxiety and potential risk and protective factors in cancer patients: a network approach. J Behav Med. (2020) 43:553–63. doi: 10.1007/s10865-019-00084-7 31435892 PMC7366596

[13] Baussard L Ernst M Diep A Jerusalem G Vanhaudenhuyse A Marie N . Network analyses applied to the dimensions of cancer-related fatigue in women with breast cancer. Cancer Med. (2024) 13:e70268. doi: 10.1002/cam4.70268 39387227 PMC11465027

[14] Li H Xiong Y Zhang Q Lu Y Chen Q Wu S . The interplay between sleep and cancer-related fatigue in breast cancer: A casual and computer-simulated network analysis. Asia Pac J Oncol Nurs. (2025) 12:100692. doi: 10.1016/j.apjon.2025.100692 40264549 PMC12013401

[15] Blanken TF Deserno MK Dalege J Borsboom D Blanken P Kerkhof GA . The role of stabilizing and communicating symptoms given overlapping communities in psychopathology networks. Sci Rep. (2018) 8:5854. doi: 10.1038/s41598-018-24224-2 29643399 PMC5895626

[16] Barth J Munder T Gerger H Nüesch E Trelle S Znoj H . Comparative efficacy of seven psychotherapeutic interventions for patients with depression: A network meta-analysis. Focus (Am Psychiatr Publ). (2016) 14:229–43. doi: 10.1176/appi.focus.140201 31997951 PMC6519647

[17] Stratton SJ . Population research: Convenience sampling strategies. Prehosp Disaster Med. (2021) 36:373–4. doi: 10.1017/s1049023x21000649 34284835

[18] Liang M Pan Y Cai J Xiong Y Liu Y Chen L . Navigating specific targets of breast cancer symptoms: An innovative computer-simulated intervention analysis. Eur J Oncol Nurs. (2025) 74:102708. doi: 10.1016/j.ejon.2024.102708 39631144

[19] Okuyama T Akechi T Kugaya A Okamura H Shima Y Maruguchi M . Development and validation of the cancer fatigue scale: a brief, three-dimensional, self-rating scale for assessment of fatigue in cancer patients. J Pain Symptom Manage. (2000) 19:5–14. doi: 10.1016/s0885-3924(99)00138-4 10687321

[20] Shun SC Beck SL Pett MA Berry PH . Psychometric testing of three Chinese fatigue instruments in Taiwan. J Pain Symptom Manage. (2006) 32:155–67. doi: 10.1016/j.jpainsymman.2006.02.011 16877183

[21] Zhang F Ding Y Han L . Reliability and validity of the Chinese version of cancer fatigue scale. Chin J Ment Health. (2011) 25:810–3. doi: 10.3969/j.issn.1000-6729.2011.11.003

[22] Buysse DJ Reynolds CF Monk TH Berman SR Kupfer DJ . The Pittsburgh Sleep Quality Index: a new instrument for psychiatric practice and research. Psychiatry Res. (1989) 28:193–213. doi: 10.1016/0165-1781(89)90047-4 2748771

[23] Liu X Tang M Hu L . Reliability and validity of the Pittsburgh sleep qualityindex. Chin J Psychiatry. (1996) 29 (2):103–7.

[24] Covic T Cumming SR Pallant JF Manolios N Emery P Conaghan PG . Depression and anxiety in patients with rheumatoid arthritis: prevalence rates based on a comparison of the Depression, Anxiety and Stress Scale (DASS) and the hospital, Anxiety and Depression Scale (HADS). BMC Psychiatry. (2012) 12:6. doi: 10.1186/1471-244x-12-6 22269280 PMC3285517

[25] Hawker GA Mian S Kendzerska T French M . Measures of adult pain: Visual Analog Scale for Pain (VAS Pain), Numeric Rating Scale for Pain (NRS Pain), McGill Pain Questionnaire (MPQ), Short-Form McGill Pain Questionnaire (SF-MPQ), Chronic Pain Grade Scale (CPGS), Short Form-36 Bodily Pain Scale (SF-36 BPS), and Measure of Intermittent and Constant Osteoarthritis Pain (ICOAP). Arthritis Care Res (Hoboken). (2011) 63:S240–52. doi: 10.1002/acr.20543 22588748

[26] Beck MW . NeuralNetTools: Visualization and analysis tools for neural networks. J Stat Softw. (2018) 85:1–20. doi: 10.18637/jss.v085.i11 30505247 PMC6262849

[27] Jones PJ Ma R McNally RJ . Bridge centrality: A network approach to understanding comorbidity. Multivariate Behav Res. (2021) 56:353–67. doi: 10.1080/00273171.2019.1614898 31179765

[28] Epskamp S Borsboom D Fried EI . Estimating psychological networks and their accuracy: A tutorial paper. Behav Res Methods. (2018) 50:195–212. doi: 10.3758/s13428-017-0862-1 28342071 PMC5809547

[29] McNally RJ . Network analysis of psychopathology: Controversies and challenges. Annu Rev Clin Psychol. (2021) 17:31–53. doi: 10.1146/annurev-clinpsy-081219-092850 33228401

[30] McNally RJ Heeren A Robinaugh DJ . A Bayesian network analysis of posttraumatic stress disorder symptoms in adults reporting childhood sexual abuse. Eur J Psychotraumatol. (2017) 8:1341276. doi: 10.1080/20008198.2017.1341276 29038690 PMC5632780

[31] Borsboom D Robinaugh DJ Rhemtulla M Cramer AOJ . Robustness and replicability of psychopathology networks. World Psychiatry. (2018) 17:143–4. doi: 10.1002/wps.20515 29856550 PMC5980315

[32] Marsman M Borsboom D Kruis J Epskamp S van Bork R Waldorp LJ . An introduction to network psychometrics: Relating Ising network models to item response theory models. Multivariate Behav Res. (2018) 53:15–35. doi: 10.1080/00273171.2017.1379379 29111774

[33] Lunansky G Naberman J van Borkulo CD Chen C Wang L Borsboom D . Intervening on psychopathology networks: Evaluating intervention targets through simulations. Methods. (2022) 204:29–37. doi: 10.1016/j.ymeth.2021.11.006 34793976

[34] Bower JE . Cancer-related fatigue--mechanisms, risk factors, and treatments. Nat Rev Clin Oncol. (2014) 11:597–609. doi: 10.1038/nrclinonc.2014.127 25113839 PMC4664449

[35] Ruiz-Casado A Álvarez-Bustos A de Pedro CG Méndez-Otero M Romero-Elías M . Cancer-related fatigue in breast cancer survivors: A review. Clin Breast Cancer. (2021) 21:10–25. doi: 10.1016/j.clbc.2020.07.011 32819836

[36] Bower JE Radin A Ganz PA Irwin MR Cole SW Petersen L . Inflammation and dimensions of fatigue in women with early stage breast cancer: A longitudinal examination. Cancer. (2025) 131:e70038. doi: 10.1002/cncr.70038 41047833 PMC12498077

[37] Klassen O Schmidt ME Scharhag-Rosenberger F Sorkin M Ulrich CM Schneeweiss A . Cardiorespiratory fitness in breast cancer patients undergoing adjuvant therapy. Acta Oncol. (2014) 53:1356–65. doi: 10.3109/0284186x.2014.899435 24837860

[38] Schmidt ME Wiskemann J Schneeweiss A Potthoff K Ulrich CM Steindorf K . Determinants of physical, affective, and cognitive fatigue during breast cancer therapy and 12 months follow-up. Int J Cancer. (2018) 142:1148–57. doi: 10.1002/ijc.31138 29082588

[39] Vannorsdall TD Straub E Saba C Blackwood M Zhang J Stearns K . Interventions for multidimensional aspects of breast cancer-related fatigue: a meta-analytic review. Support Care Cancer. (2021) 29:1753–64. doi: 10.1007/s00520-020-05752-y 33089371

[40] Schmidt ME Wiskemann J Armbrust P Schneeweiss A Ulrich CM Steindorf K . Effects of resistance exercise on fatigue and quality of life in breast cancer patients undergoing adjuvant chemotherapy: A randomized controlled trial. Int J Cancer. (2015) 137:471–80. doi: 10.1002/ijc.29383 25484317

[41] Chang Z Zhang Y Liang X Chen Y Guo C Chi X . A network analysis of depression and anxiety symptoms among Chinese elderly living alone: based on the 2017-2018 Chinese Longitudinal Healthy Longevity Survey (CLHLS). BMC Psychiatry. (2025) 25:28. doi: 10.1186/s12888-024-06443-2 39780094 PMC11716466

[42] Dinapoli L Colloca G Di Capua B Valentini V . Psychological aspects to consider in breast cancer diagnosis and treatment. Curr Oncol Rep. (2021) 23:38. doi: 10.1007/s11912-021-01049-3 33709235 PMC7952347

[43] Berry RB Budhiraja R Gottlieb DJ Gozal D Iber C Kapur VK . Rules for scoring respiratory events in sleep: update of the 2007 AASM manual for the scoring of sleep and associated events. Deliberations of the Sleep Apnea Definitions Task Force of the American Academy of Sleep Medicine. J Clin Sleep Med. (2012) 8:597–619. doi: 10.5664/jcsm.2172 23066376 PMC3459210

[44] Åkerstedt T Hume K Minors D Waterhouse J . The meaning of good sleep: a longitudinal study of polysomnography and subjective sleep quality. J Sleep Res. (1994) 3:152–8. doi: 10.1111/j.1365-2869.1994.tb00122.x 10607120

[45] Hasler BP Troxel WM . Couples' nighttime sleep efficiency and concordance: evidence for bidirectional associations with daytime relationship functioning. Psychosom Med. (2010) 72:794–801. doi: 10.1097/PSY.0b013e3181ecd08a 20668283 PMC2950886

[46] Savitz DA Wellenius GA . Can cross-sectional studies contribute to causal inference? It depends. Am J Epidemiol. (2023) 192:514–6. doi: 10.1093/aje/kwac037 35231933

