# Supplementary material for: Navigating specific targets of psychoneurological symptom cluster in breast cancer: a computer-simulated network analysis
Source: Front Oncol. 2026 Jun 26;16:1869133. doi: 10.3389/fonc.2026.1869133 (PMC13351552; doi:10.3389/fonc.2026.1869133)
Supplement: Supplementary file 1 [file DataSheet1.docx]

**Supplementary Materials**

**Table S1 Edge weights with 95% CIs in Gaussian network analysis**

**Figure S1 Bootstrapped stability test for expected influence**

**Figure S2 Accuracy estimations of all edge weights using the non-parametric bootstrapping method**

**Table S1 Edge weights with 95% CIs in Gaussian network analysis**

| **Edge** | **Weight** | **Lower 95%CI** | **Upper 95%CI** |
| --- | --- | --- | --- |
| AX--DP | 0.702 | 0.616 | 0.747 |
| S3--S4 | 0.428 | 0.314 | 0.497 |
| S1--S2 | 0.368 | 0.262 | 0.445 |
| PF--EF | 0.349 | 0.246 | 0.412 |
| PF--CF | 0.304 | 0.175 | 0.397 |
| PF--PA | 0.273 | 0.181 | 0.344 |
| S2--S3 | 0.187 | 0.095 | 0.280 |
| PF--S7 | 0.157 | 0.079 | 0.236 |
| S2--S4 | 0.156 | 0.028 | 0.246 |
| S2--S5 | 0.148 | 0.042 | 0.224 |
| CF--S7 | 0.146 | 0.048 | 0.229 |
| S1--S3 | 0.137 | 0.025 | 0.226 |
| S1--S6 | 0.133 | 0.000 | 0.202 |
| S7--PA | 0.131 | 0.022 | 0.221 |
| PF--S1 | 0.127 | 0.038 | 0.196 |
| DP--S7 | 0.112 | 0.000 | 0.171 |
| AX--S1 | 0.104 | 0.017 | 0.160 |
| S5--S7 | 0.103 | 0.000 | 0.179 |
| S1--S7 | 0.093 | 0.000 | 0.186 |
| EF--DP | 0.086 | 0.006 | 0.151 |
| S2--S7 | 0.078 | 0.000 | 0.171 |
| CF--PA | 0.067 | 0.000 | 0.152 |
| S1--S5 | 0.065 | 0.000 | 0.145 |
| AX--PA | 0.048 | 0.000 | 0.117 |
| PF--S5 | 0.044 | 0.000 | 0.123 |
| EF--PA | 0.042 | 0.000 | 0.134 |
| CF--S5 | 0.036 | 0.000 | 0.119 |
| PF--DP | 0.036 | 0.000 | 0.103 |
| DP--PA | 0.035 | 0.000 | 0.133 |
| S3--S7 | 0.035 | 0.000 | 0.125 |
| S2--S6 | 0.032 | 0.000 | 0.106 |
| S3--S6 | 0.030 | 0.000 | 0.123 |
| PF--AX | 0.030 | 0.000 | 0.105 |
| S4--S6 | 0.027 | 0.000 | 0.109 |
| CF--DP | 0.019 | 0.000 | 0.088 |
| EF--S2 | -0.017 | -0.084 | 0.000 |
| S4--S7 | 0.014 | 0.000 | 0.089 |
| S1--S4 | 0.008 | 0.000 | 0.097 |
| S5--PA | 0.006 | 0.000 | 0.074 |
| AX--S7 | 0.003 | 0.000 | 0.095 |
| AX--S2 | 0.000 | 0.000 | 0.050 |
| AX--S3 | 0.000 | -0.035 | 0.000 |
| AX--S4 | 0.000 | 0.000 | 0.021 |
| AX--S5 | 0.000 | 0.000 | 0.016 |
| AX--S6 | 0.000 | 0.000 | 0.075 |
| CF--AX | 0.000 | 0.000 | 0.056 |
| CF--S1 | 0.000 | 0.000 | 0.059 |
| CF--S2 | 0.000 | 0.000 | 0.003 |
| CF--S3 | 0.000 | -0.044 | 0.000 |
| CF--S4 | 0.000 | 0.000 | 0.058 |
| CF--S6 | 0.000 | -0.087 | 0.018 |
| DP--S1 | 0.000 | 0.000 | 0.055 |
| DP--S2 | 0.000 | 0.000 | 0.027 |
| DP--S3 | 0.000 | 0.000 | 0.017 |
| DP--S4 | 0.000 | 0.000 | 0.028 |
| DP--S5 | 0.000 | -0.007 | 0.019 |
| DP--S6 | 0.000 | -0.016 | 0.004 |
| EF--AX | 0.000 | 0.000 | 0.000 |
| EF--CF | 0.000 | 0.000 | 0.111 |
| EF--S1 | 0.000 | 0.000 | 0.028 |
| EF--S3 | 0.000 | -0.010 | 0.019 |
| EF--S4 | 0.000 | -0.002 | 0.015 |
| EF--S5 | 0.000 | -0.055 | 0.028 |
| EF--S6 | 0.000 | 0.000 | 0.025 |
| EF--S7 | 0.000 | 0.000 | 0.000 |
| PF--S2 | 0.000 | 0.000 | 0.036 |
| PF--S3 | 0.000 | -0.021 | 0.000 |
| PF--S4 | 0.000 | 0.000 | 0.055 |
| PF--S6 | 0.000 | -0.054 | 0.000 |
| S1--PA | 0.000 | 0.000 | 0.079 |
| S2--PA | 0.000 | 0.000 | 0.020 |
| S3--PA | 0.000 | -0.061 | 0.000 |
| S3--S5 | 0.000 | 0.000 | 0.068 |
| S4--PA | 0.000 | 0.000 | 0.007 |
| S4--S5 | 0.000 | 0.000 | 0.065 |
| S5--S6 | 0.000 | 0.000 | 0.088 |
| S6--PA | 0.000 | -0.057 | 0.000 |
| S6--S7 | 0.000 | 0.000 | 0.075 |


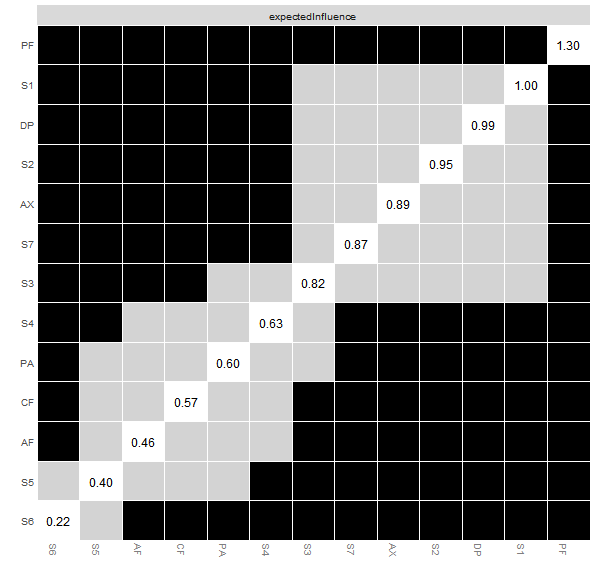


**Figure S1 Bootstrapped stability test for expected influence**


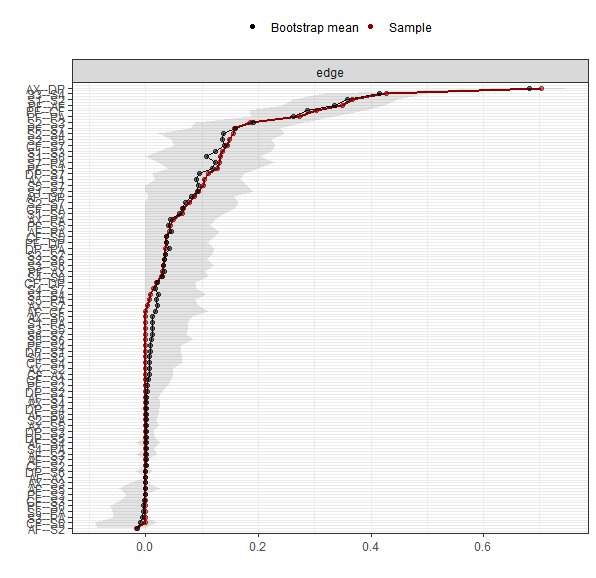


**Figure S2 Accuracy estimations of all edge weights using the non-parametric bootstrapping method**
